# Bruton’s Tyrosine Kinase: A Potential Novel Target for Neurological Disorders

**DOI:** 10.33549/physiolres.935494

**Published:** 2025-04-01

**Authors:** Debanjan DAS, Arijit GHOSH, Denise GRECO, Danica MICHALIČKOVÁ, Ondřej SLANAŘ

**Affiliations:** 1 Institute of Pharmacology, First Faculty of Medicine, Charles University and General University Hospital in Prague, Prague, Czech Republic; 2 School of Life Sciences and Biotechnology, Shanghai Jiao Tong University, Shanghai, China; 3 Department of Physiology, Faculty of Science, Charles University, Prague, Czech Republic

**Keywords:** Alzheimer’s disease, Brain injury, Bruton’s tyrosine kinase, Multiple sclerosis, Neuroinflammation, NLRP3, PCNSL

## Abstract

Bruton’s tyrosine kinase (BTK) is a crucial part of the B-cell receptor signaling pathway that has been extensively studied in various types of malignancies. Recent studies have extended our knowledge on its role in metabolism as well as neurological disorders. It may play an important role in the pathophysiology of neurological diseases, such as multiple sclerosis, Alzheimer’s disease, brain injury, and several others. Activation of inflammasomes, mainly NLRP3, is one of the core mechanisms by which it promotes inflammation in the brain related to aging and diseases. In this paper, we provide an overview of the less explored roles of BTK in several brain diseases and discuss the potential of its inhibition to become a therapeutic target for neurological diseases.

## Background

Bruton’s tyrosine kinase (BTK) is a 659 amino acid-long protein belonging to one of the five members of the Tec family. It facilitates B-cell receptor signaling, which is responsible for all aspects of the B-cell life cycle, including proliferation, maturation, and apoptosis [1]. It is expressed in blood stem cells, hematopoietic cells, macrophages, microglia, neutrophils and mast cells but not in T and plasma cells [2]. Mast cell and basophil Fc receptor (FcR) signaling as well as macrophage FcR signaling are also regulated by BTK. Downstream signaling stimulates the expression of proinflammatory cytokines, chemokines, and cell adhesion molecules when BTK is activated through Fc and FcRs [3]. Mutation of the BTK gene results in X-linked agammaglobulinemia (XLA) in humans, which is a rare immunodeficiency that prevents the development of mature B lymphocytes, leading to agammaglobulinemia. Owing to its key role, BTK has become a very appealing pharmacological target for autoimmune and inflammatory disorders, chronic lymphocytic leukemia, mantle cell lymphoma, and other B-cell malignancies [4]. Interestingly, a recent clinical trial indicated that BTK inhibition decreases disease flares in multiple sclerosis patients [5], but the evidence for the role of BTK in autoimmune and inflammatory disorders is sporadic [6].

## BTK structure and its inhibitors

Structurally, BTK consists of five domains, starting from the N-terminus toward the C-terminus: the pleckstrin homology (PH) domain, the proline-rich Tec homology (TH) domain, the sarcoma (Src) homology (SH) domains (named SH3 and SH2), and the catalytic domain. The PH domain promotes protein-phospholipid and protein–protein interactions, while the TH domain contains a zinc finger motif that is essential for protein stability and function. The catalytic domain’s Tyr551 and Cys481 phosphorylation sites are the targets of irreversible inhibitors, while the SH2 and SH3 domains’ Tyr223 is the autophosphorylation site [7].

**Fig. 1:**
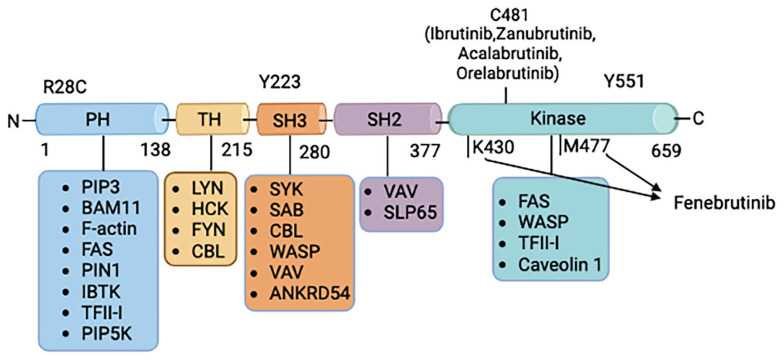
Structure of BTK and its interactions with other genes. BTK consists of 659 amino acids and contains five domains, from the N-terminus to the C-terminus; the domains are listed as the pleckstrin homology (PH) domain, proline-rich Tec homology (TH) domain, Src homology (SH) domain SH3, SH2, and catalytic domain. Four approved BTK inhibitors by the FDA mainly target the catalytic domain of BTK. Irreversible BTK inhibitors bind to C481, while reversible BTK inhibitors do not bind to C481, such as fenebrutinib, which forms hydrogen bonds with K430 and M477.

On the basis of the mechanism of action, BTKi can be divided into two categories: 1) irreversible inhibitors, which bind to the Cys-481 residue of BTK, and 2) reversible inhibitors, which bind to some specific pockets of the SH3 domain (Fig. 1.) [8]. All existing BTKi that can be found on the market are irreversible inhibitors, although some reversible inhibitors have been patented and are currently under preclinical or clinical investigation for autoimmune diseases such as MS [9,10].

### Irreversible inhibitors

All the inhibitors currently approved by major regulatory agencies are irreversible inhibitors of BTK; the first inhibitor, known as ibrutinib, was approved in 2013. Later, acalabrutinib and zanubrutinib were approved in 2017 and 2019, respectively. In Japan, tirabrutinib is approved by the Pharmaceutical and Medical Device Agency (PMDA, Japan) for the treatment of Waldenström macroglobulinemia, lymphoplasmacytic lymphoma, and beta cell lymphoma. In December 2020, the Chinese Food and Drug Administration gave its first approval for orelabrutinib to be used in mantle cell lymphoma, chronic lymphocytic leukemia, and small lymphocytic lymphoma in China for recurring treatment.

The currently available inhibitors not only inhibit BTK but also block other kinases with Cys-481-like residues, such as Tec, IL-2-inducible tyrosine kinase (ITK), and B-lymphoid tyrosine kinase (BLK) [11]. Such a lack of selectivity results in a high occurrence of several adverse effects, such as fever, edema, diarrhea, and bleeding.

The IC_50_ of the irreversible BTKi generally ranges in the low nanomolar range. There are 4 major sites for irreversible binding of BTKi: i) a large hydrophobic group, ii) an aromatic heterocyclic nucleus, iii) a warhead terminal group, and iv) a linker. The linker generally connects the warhead terminal group to the aromatic heterocyclic nucleus, facilitating a covalent bond with Cys481 [12,13].

#### - First generation of irreversible BTKi

**Fig. 2:**
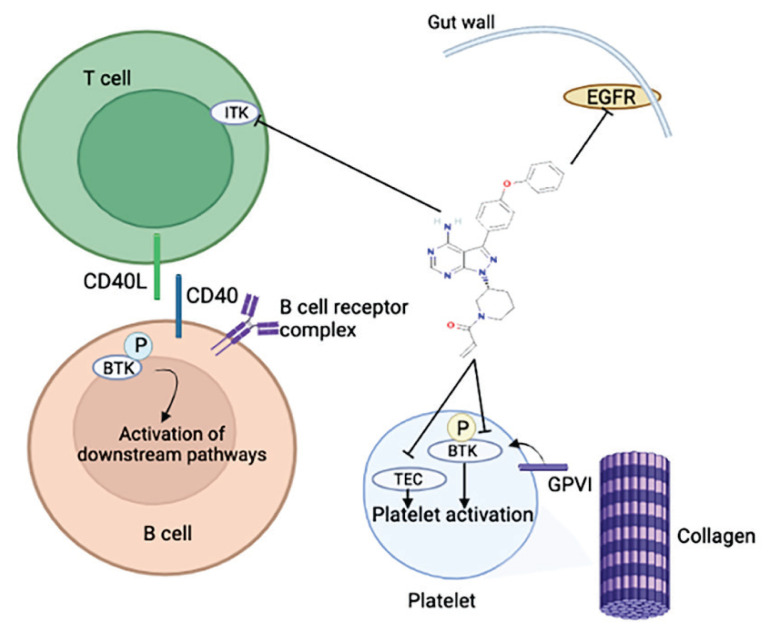
Actions of ibrutinib on different cell types. Ibrutinib inhibits the phosphorylation of BTK, thereby inhibiting the activation of downstream signaling pathways (solid black lines). Ibrutinib shows off-target effects through the inhibition of additional kinases, including Tec, ITK, and EGFR. BTK, Bruton tyrosine kinase; EGFR, epidermal growth factor receptor; GpvI, glycoprotein VI; ITK, interleukin-2-inducible T-cell kinase; TEC, tyrosine kinase expressed in hepatocellular carcinoma.

Ibrutinib (formerly PCI-32765) is an irreversible selective small molecule BTKi with the ability to form a covalent bond with Cys-481 in the ATP-binding domain of BTK, resulting in an IC_50_ of 0.5 nM [14]. Ibrutinib was first approved in the USA for use in refractory mantle cell lymphoma in 2013 [15], chronic lymphocytic leukemia in 2014 [16], Waldenström’s macroglobulinemia in 2015 [17], marginal zone lymphoma [13], chronic graft-versus-host disease and allogeneic hematopoietic cell transplantation (allo-HCT) in 2017 [18]. Ibrutinib blocks both BTK and the Tec family of kinases. These kinases possess major roles in B and T-cell signaling along with their involvement in the platelet activation pathway via collagen receptor glycoprotein VI (Fig. 2.) [19].

Ibrutinib possesses a risk of adverse effects such as diarrhea, fatigue, bleeding (hemorrhage), hyperlipidemia, ventricular arrhythmia, atrial fibrillation and atrial flutter. It may also cause a decrease in blood cell count. It has been shown that most of its adverse effects are between grade 1 and 2, but grade 3 may develop in approximately 2 % of patients [20]. In a long-term phase 3 study of ibrutinib in patients with relapsed/refractory CLL/SLL over 71 months, 16 % of patients experienced an adverse effect of either grade and had to discontinue ibrutinib [21]. A major toxicity of ibrutinib is bleeding, which occurs due to prevention of platelet aggregation and inhibition of glycoprotein VI (GPVI) signaling in patients with X-linked agammaglobulinemia [22].

#### - Second generation of irreversible BTKi

Despite the remarkable efficacy of the first generation, their adverse effects and disease resistance to treatment made the urge to consider beyond ibrutinib. These obstacles led to the development of more specific BTKi, which resulted in development of the second generation.

Acalabrutinib and zanubrutinib are second-generation BTK inhibitors that were approved by the Food and Drug Administration (FDA) in July 2020 and by the European Medicines Agency (EMA) in November 2021, respectively. These drugs are marketed as Calquence® and Brukinsa®, respectively, and are used to treat various types of cancers, such as chronic lymphocytic leukemia, mantle cell lymphoma, marginal zone lymphoma, and Waldenström macroglobulinemia. They are preferred over the first-generation drugs because of their better safety profile and higher efficacy [23–25]. In a comparative clinical study, zanubrutinib showed better efficacy and lower cardiac toxicity than first-generation BTKi [26]. Acalabrutinib binds to Cys-481, similar to ibrutinib, but with improved selectivity and fewer adverse effects. In a comparative clinical trial in patients with non-Hodgkin lymphoma (NHL) with ibrutinib and acalabrutinib, it was found that acalabrutinib does not inhibit Src family kinases, which play a major role in platelet adhesion to collagen [27,28].

### Reversible inhibitors

Noncovalent (reversible) BTKis have a number of benefits over the currently approved covalent inhibitors due to their better safety profile. Noncovalent inhibitors can also be useful where malignant cells have developed resistance against ibrutinib treatment, as they can bind to C481S and C481R BTK variants and induce potential treatment effects. Along with drug resistance, reversible inhibitors exhibit fewer adverse effects owing to their modified chemical structures, which are different from the irreversible BTKi, and can effectively prevent whole blood beta-cell activation [29].

In this thorough analysis, twelve BTKi (Table 1) approved or currently being investigated in clinical trials are discussed. Special attention was given to their BTK-selectivity, pharmacokinetic and pharmacodynamic characteristics, disease targeting characteristics, adverse effects such as bleeding, and effects on platelets.

The discovery of further reversible inhibitors will depend on many factors, such as an enhanced selectivity profile, reduced adverse effects and higher potency than others [30].

## BTK in the signaling of immune cells

BTK is a member of the Tec family of nonreceptor protein tyrosine kinases, which are involved in the signaling pathways of various immune cells, such as B cells and myeloid cells (including microglia, macrophages, monocytes, mast cells, basophils, neutrophils, and dendritic cells). Other members of the Tec family include IL-2-inducible T-cell kinase, resting lymphocyte kinase, TEC, and bone marrow-expressed kinase [31]. The presence of a PH domain is a key feature of Tec family proteins, allowing them to bind to phosphoinositides, such as phosphatidylinositol (3,4,5)-trisphosphate (PtdIns(3,4,5)P3), in the cyto-plasmic leaflet of the plasma membrane. When the membrane phosphoinositide concentration increases due to receptor activation, Tec kinases are recruited to the plasma membrane, where they phosphorylate downstream signaling molecules. The PH domain is essential for controlling downstream effectors that regulate cell growth, differentiation, metabolism, survival, and apoptosis [32–34].

BTK plays a critical role in the development and maturation of B cells in various parts of the body, including the bone marrow, secondary lymphoid tissues, and the periphery. It regulates the transition of pre-B cells into immature B cells by controlling the proliferation of large pre-B cells induced by IL-7, as well as their transition from cycling to resting states [35,36]. In humans, mutations in BTK can cause X-linked agammaglobulinemia (XLA), which affects B-cell and plasma cell levels in the blood. XLA patients typically have a significant reduction in circulating B cells, with less than 2 % of total lymphocytes being B cells [37,38].

Beyond the bone marrow, BTK is involved in the migration and maturation of B cells in follicles by regulating integrin gene expression. Furthermore, it controls the activation and final differentiation of B cells into plasma cells or memory B cells [39–41].

Aside from its role in B-cell signaling, BTK also has significant signaling functions in macrophages through the IgG-specific Fc receptor (FcγR), as well as in mast cells via the IgE-specific Fc receptor (FcɛR). BTK regulates degranulation, the release of histamine and proinflammatory cytokines, the production of reactive oxygen species (ROS), and the activation of the inflammasome in myeloid cells by interacting with these Fc receptors [42,43].

**Fig. 3:**
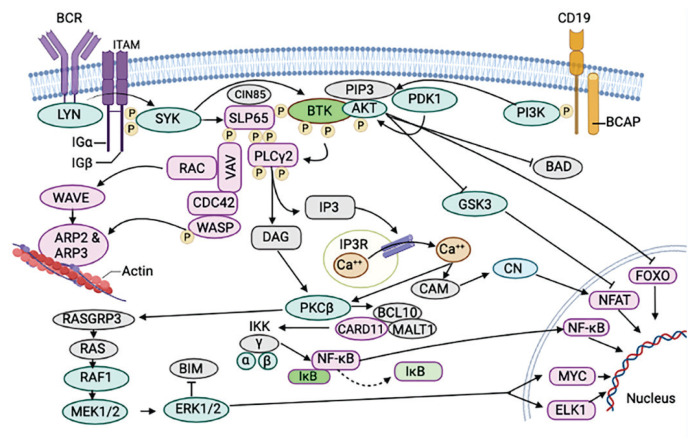
Association of BTK in B-cell receptor signaling. The creation of a microsignalosome made up of VAV, phosphoinositide 3-kinases (PI3K), BTK, leukocyte protein of 65 kDa (SLP65), and phosphor-lipase C2 (PLC2) is the outcome of B-cell receptor (BCR) signaling. BTK is primarily in charge of activating PLC2, which triggers an influx of Ca^2+^, the transcription factors nuclear factor-KB (NF-KB) and nuclear receptor of activated T cells (NFAT), and extracellular signal-regulated kinase (ERK1 or ERK2) activation. In addition, BTK activation causes the Wiskott-Aldrich syndrome protein (WASP) to become active, which results in cytoskeleton alterations linked to BCR activation. Ak strain trans-forming (AKT) is triggered by PI3K, which phosphorylates (P) forkhead box O (FOXO) transcription factors and renders them inactive, independent of Ca^2+^. AKT releases the BH3-only protein BCL-2 antagonist of cell death (BAD) from BCL-XL and stabilizes MCL1 by blocking proapoptotic proteins, including AKT. Actin-related protein, B-cell adapter for PI3K, BCL-2 interacting mediator of cell death. CARD11, protein 11 with a caspase recruitment domain; CIN85, an 85 kDa CBL-interacting protein; diacylglycerol, glycogen synthase kinase, calcineurin, and immunoglobulin, NF-KB kinase inhibitor, and Ig (immunoglobulin) IP3, phosphatidylinositol-3,4,5, trisphosphate; IP3R, the IP3 receptor; ITAM, the immunoreceptor tyrosine-based activation motif; MALT1, the mucosa-associated lymphoid tissue lymphoma translocation protein 1; PDK1, the 3-phosphoinositide-dependent protein kinase 1; PIP3, the phosphatidylinositol-3,4,5.

Fig. 3. depicts the association of BTK in B-cell receptor signaling.

## Inflammation and autoimmunity as key links for the action of BTK inhibitors in the brain

Inflammation of the brain (neuroinflammation) has been established as one of the crucial mechanisms in brain diseases, and molecules targeted at neuroinflammation have been of core interest as therapeutic targets in neurological disorders. It refers to the inflammatory reaction generated by cells residing in the central nervous system (CNS) as well as cells from outside the CNS, occurring in both the brain and the spinal cord after an injury or disturbance. Glial cells, including microglia, astrocytes, endothelial cells, and peripheral immune cells, such as macrophages, produce cytokines, chemokines, ROS, and secondary messengers under inflammatory conditions. These insults can be either beneficial or harmful, depending on the degree of responses produced and the type of molecules liberated [44]. Although a low degree of immune responses has beneficial roles in brain development and in the regulation of memory and learning, sustained responses with a higher degree of neuroinflammation may cause neuronal damage/apoptosis, neurodegeneration, increased aging, and anxiety and depression [45,46].

In the brains of patients with Alzheimer’s disease (AD), for example, abnormal microglial activity is one of the causes of synaptic loss. In postmortem AD brains, microglia surrounding amyloid plaques express IL-1 [47], which is one of the downstream targets in the BTK-related pathway. In both the mouse and human brain, the BTK transcript is found at high levels in microglial cells compared to other cell types [48,49]. Protein levels of BTK are highly expressed in microglia, while blocking BTK with an experimental inhibitor (CC-292) or ibrutinib causes a reduction in phagocytosis of synaptic structures by microglia [50]. Ibrutinib also reduces the overall activation of microglia, a characteristic of inflammatory reactions in the brain, in AD mouse models [51]. Similarly, targeting microglia through BTK could hold great potential against multiple sclerosis (MS) [52].

One of the mechanisms by which BTK induces neuroinflammation involves activation of the NLRP3 inflammasome. This is the most studied inflammasome in human diseases and has been shown to be associated with MS, AD, Parkinson’s disease, obesity, type 2 diabetes, and atherosclerosis [53]. Activation of NOD-, LRR- and pyrin domain-containing protein 3 (NLRP3) takes place through its priming by binding of LPS to TLR4, while cellular expression of the inflammasome is increased through signaling pathways such as NF-κB signaling [54]. It controls caspase-1 activation, which is required for the secretion of IL-1β and IL-18, and has been shown to control the aging process in the periphery. Additionally, age-related hippocampal astrogliosis and inflammation in the CNS and a reduction in the inflammasome have been reported to reduce age-associated innate immune activation [55].

Ito *et al*. (2015) and Jin *et al*. (2021) showed that BTK is essential for NLRP3 activation in the context of ischemic brain injury [56,57]. The infarct region reportedly had more BTK^+^ cells that were negative for microtubule-associated protein 2 (MAP2), while these cells were also positive for active caspase-1 and NLRP3, suggesting that BTK and inflammasomes are activated in infiltrating macrophages and/or microglia in the infarct area [57]. As shown in *Btk* knockout (KO) mice and *in vitro* human and murine cell lines experiments, BTK regulates NLRP3 inflammasome activity through a phospho-tyrosine switch that helps in the relocalization and oligomerization of NLRP3 and promotes full inflammasome assembly by regulating other inflammasomes, such as the polymerization of apoptosis-associated speck-forming protein (ASC). Phosphorylation of BTK upon NLRP3 activation also suggests that its kinase activity might be crucial for the NLRP3 activation process [58]. Phosphorylation of BTK at Tyr223 within the SH3 domain is necessary for the full activation of BTK and its downstream signaling, including induction of the NLRP3 inflammasome, as observed in a murine models of stress [59]. In a transient middle cerebral artery occlusion model of ischemic injury, BTK as well as NLRP3 along with their downstream cytokine IL-1β were found to be increased. Moreover, BTK was found to be colocalized with NLRP3 in postischemic neurons, suggesting their joint effects in neuronal injury mediated by neuroinflammation [60]. Similarly, in mouse models of stress, the preclinical efficacy of BTKi has been reported to be linked to NLRP3 through the downregulation of caspase-1 and IL-1β [61]. Although the current evidence does not link BTK with NLRP3 in the pathophysiology of MS/AD, it is easy to infer the same, given the link between NLRP3 and these diseases [62,63] and taking into account the efficacy of BTKi in these diseases.

**Fig. 4:**
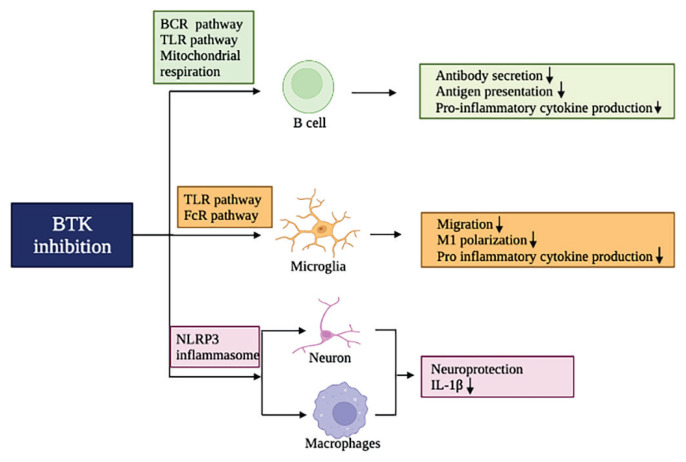
Overview of immuno-modulatory mechanisms related to BTK inhibition. When BTK is inhibited within the CNS, it has various effects on the immune system. In B cells, inhibition of BTK reduces the ability to present antigens, produce antibodies, and generate proinflam-matory cytokines through pathways such as the BCR and TLR pathways. Additionally, BTK inhibition can decrease the proinflammatory profile, migration, and cytokine production of microglia. BTK inhibition also prevents the activation of the NLRP3 inflammasome in neurons, macro-phages, and neutrophils, which leads to a decrease in IL-1β production and ultimately provides neuroprotection.

In addition to NLRP3, BTK inhibition affects several other pathways that regulate immune responses and inflammatory reactions (Fig. 4.).

## BTK and its inhibitors in neurological disorders

### Multiple sclerosis

MS is a chronic inflammatory disease of the central nervous system (CNS) that causes persistent demyelination and neurodegeneration with incompletely elucidated etiology or definitive treatment. The myelin sheaths that encase neurons are harmed in MS by a complicated interaction of immunological and neurodegenerative events. Relapsing-remitting MS (RRMS), primary progressive MS (PPMS), and secondary progressive MS (SPMS) are the three main clinical subtypes of MS. Clinical relapses, during which immune cells invade the CNS and generate focal lesions that may be seen by MRI, are a hallmark of RRMS. Progressive versions of the disease (PPMS and SPMS) are thought to be more neurodegenerative in nature and to involve less immune activation and inflammation [64].

Once, T cells were seen as the main drivers of the autoimmune response in MS. Autoreactive Th1 and Th17 cells activated in peripheral lymph nodes migrate into the CNS by crossing the blood–brain barrier, where they are reactivated. By secreting proinflammatory cytokines, these cells drive the functions of CNS-resident cells (microglia, astrocytes, oligodendrocytes). Nevertheless, the background of MS pathophysiology is more complicated and involves more types of leukocytes, including B cells, T regulatory cells, and myeloid cells. Previously, B cells had been seen as a relatively uniform and passive population, which need the help of T cells to differentiate into plasmablasts and plasma cells secreting CNS-autoreactive antibodies. However, new emerging data acknowledge a much broader range of B-cell functions in MS pathology, including activation of T cells and autoantigen targeting, production of proinflammatory cytokines (IL-6, tumor necrosis factor-α (TNF), lymphotoxin-α (LT-α) and granulocyte-macrophage colony stimulating factor (GM-CSF), generation of ectopic germinal centers, and antibody production [65]. A recent study highlights the role of B-cell secreted products in MS, demonstrating their potential contribution to hallmark features of subpial cortical injury, including demyelination, neuronal loss, and microglial activation. The findings suggest that these products may activate both resident microglia and infiltrating macrophages, contributing to acute and chronic MS lesions. Additionally, the research reveals a bi-directional interaction between pro-inflammatory B cells and myeloid cells, potentially propagating CNS inflammation and injury. Conversely, IL-10 expressing B cells are shown to promote anti-inflammatory responses, offering therapeutic opportunities to mitigate CNS-compartmentalized inflammation. Strategies include limiting pro-inflammatory B-cell and myeloid interactions or shifting B-cell profiles toward anti-inflammatory responses. CNS-penetrating BTK inhibitors have been highlighted as promising therapeutic agents targeting both B cells and myeloid cells [66].

In line with the importance of B cells in the pathophysiology of MS, there are observations in human tissue that suggest that BTK plays a significant role in MS, particularly in progressive forms of the disease [67]. In patients with RRMS the inhibition of BTK by using BTK inhibitor reduce the enhancing lesions compared with placebo group [5].

Several BTK inhibitors have been tested in preclinical models of MS. Evobrutinib has recently undergone testing as a monotherapy for RRMS (Table 2). Three doses of evobrutinib (25 mg once daily, 75 mg once daily, and 75 mg twice daily) were compared to a placebo or dimethyl fumarate in this double-blind, randomized phase II clinical trial. A total of 267 people were enrolled in the trial. The total number of gadolinium (Gd+)-enhancing lesions on T1-weighted magnetic resonance imaging detected at weeks 12, 16, 20, and 24 served as the primary end point. The expanded disability status scale alterations from baseline and the annualized relapse rate were secondary endpoints. Patients who received 75 mg of ibrutinib once or twice per day showed a decrease in both the overall number of Gd+ lesions and ARR, according to the trial. However, statistical significance was observed only for the quantity of Gd+ lesions when placebo and 75 mg of evobrutinib were compared. In this trial, no change in the expanded disability status scale was seen. It should be noted that evobrutinib at moderate and high doses was linked to an increase in liver aminotransferase levels [5]. Larger trials are required to fully evaluate the efficacy and safety of this therapy in MS because of the uncertainties surrounding the clinical outcomes, dosing regimen, and potential adverse effects [68–70].

Currently, other two BTKi, tolebrutinib and fenebrutinib are being investigated in clinical trails (NCT04458051 and NCT04586010, respectively).

### Brain aging, dementia and Alzheimer’s disease

AD is the most common form of dementia, affecting millions of people globally. It has no definite treatment options, while the current available therapies only control the symptoms [45]. Aging is the main risk factor for dementia-related neurodegeneration and AD, and changes in the timing or nature of the cellular hallmarks of normal aging might be key to understanding the events that convert normal aging into neurodegeneration [71]. Aging causes shrinkage of the brain and changes in the vasculature at all levels, from molecular to cellular to morphological levels, resulting in cognitive decline [72]. The primary distinguishing characteristics of AD are the buildup of toxic amyloid fragments in senile plaques outside of cells, the accumulation of hyperphosphorylated tau protein in neurofibrillary tangles inside cells, and chronic inflammation in affected areas of the brain. The disease’s etiology is the subject of various theories, including the cholinergic hypothesis, amyloid hypothesis, and tau hypothesis. The amyloid hypothesis, which suggests that the abnormal processing and/or systemic clearance of β-amyloid (Aβ) are responsible for AD progression, is the most extensively researched area. Overproduction of Aβ is more frequent in familial AD, whereas sporadic AD often displays impaired Aβ clearance rather than overproduction. These changes in Aβ homeostasis lead to the formation of Aβ senile plaques, resulting in chronic activation of microglia and astrocytes, neurite damage, neurodegeneration, depletion of neurotransmitters, and the appearance of AD symptoms [45].

In a model of aging mice (*Zmpste24−/−*), BTK was shown to be overexpressed in the brains of these mice (lower expression of BTK at 3 weeks vs 70 weeks). Inhibition of BTK with ibrutinib decreased the levels of p53, p21, and p16, markers of senescence, in brain samples. However, ibrutinib treatment did not affect the markers of DNA damage, suggesting that the inhibitor prevents the onset of senescence without preventing DNA damage [73]. Interestingly, anxiety-like behaviors, factors that are associated with both aging and AD [74], have been shown to be reduced in *Zmpste24−/−* mice treated with ibrutinib. Memory loss, which is a sign of aging and aging-associated diseases such as AD, was also shown to be ameliorated in these animals following ibrutinib treatment [73]. One study reported that BTK transcripts were elevated in regions of human AD brains, and BTK expression was found to be elevated in mouse models of AD through mechanisms related to synaptic loss, which could be reversed by treatment with BTK inhibitors [50]. BTK’s importance in the removal of tau protein through the proteasome has been proposed, and the abnormal phosphorylation of tau is linked to the underlying mechanisms of AD [75]. Ibrutinib was indeed shown to be effective in a model of familial AD. In the 5xFAD mouse model of familial AD, the drug was shown to improve long-term memory and promote the spawning of spines while reducing amyloid beta accumulation and tau-related CDK5 kinase phosphorylation, which are important hallmarks of AD [51] (Table 2).

There is limited research on the application of BTKi in other forms of dementia, such as frontotemporal dementia or vascular dementia. The role of BTK in neuroinflammation suggests potential therapeutic avenues for various neurodegenerative conditions, but specific studies targeting non-AD are scarce. Further research is needed to explore the efficacy and safety of BTK inhibitors across different types of dementia beyond AD.

### Other neurological diseases

Primary central nervous system lymphoma (PCNSL) is a rare and aggressive form of diffuse large B-cell lymphoma that affects the CNS, including the brain, eyes, cerebrospinal fluid, and spinal cord [76,77]. It is often associated with mutations in the BCR subunit CD79B and the Toll-like receptor adaptor protein MYD88, which cause chronic activation of the NF-κB signaling pathway and promote the malignant proliferation of B cells [78]. BTK inhibitors such as ibrutinib, which have excellent bioavailability in terms of brain distribution, have been proposed as a promising therapeutic approach for PCNSL [79–81]. Ibrutinib was the first BTK inhibitor to be evaluated in PCNSL trials and has shown impressive clinical activity and safety in several studies [81–83]. However, resistance to ibrutinib has been observed due to mutations of two signaling subunits of BCR, CARD11 and CD79B, and combination therapy with other drugs such as rituximab and methotrexate may be necessary for ibrutinib resistance [82,84,85]. Other BTK inhibitors, such as tirabrutinib and orelabrutinib, are also being investigated in clinical trials and have shown promising results in combination with other drugs [86–88]. Overall, BTK inhibitors have shown potential as a therapeutic option for PCNSL, and further clinical studies are warranted to evaluate their efficacy and safety.

Several other studies have highlighted possible roles of this kinase in other brain diseases as well. In a model of ischemic brain injury, ibrutinib was shown to reduce infarct size and improve neurological deficits [57]. Similarly, the drug was shown to reduce lesion volume and improve functional outcomes in rats after traumatic spinal cord injury. Basso, Beattie and Bresnahan (BBB) scoring for locomotor activity revealed that rats treated with ibrutinib had higher recovery scores than vehicle-treated animals when the drug was given for 7 days and followed up to a period of 21 days. However, a longer treatment regimen for 14 days did not show any further improvement, suggesting that the drug is able to improve the functional features already at the acute stages of the treatment regimen [89].

Moreover, BTK also serves as a therapeutic target for alleviating neuromyelitis optica spectrum disorder (NMOSD) pathology. NMOSD is an auto-immune condition of the CNS, involving B-cell receptor signaling and astrocyte–microglia interactions. A recent study [90] identified increased BTK expression in the blood and cerebrospinal fluid of NMOSD patients. In an NMOSD mouse model, zanubrutinib, a specific BTKi, mitigated demyelination, edema, axonal injury, and astrocyte–microglia interaction. Additionally, reduced B-cell activation, maturation, and aquaporin-4 autoantibody production were observed. Transcriptome analysis showed that BTK inhibition downregulated chemokine-related genes and genes associated with cell adhesion and migration in microglia. Safety and efficacy of zanubrutinib are currently being evaluated in an open-label clinical study for patients with NMOSD (NCT05356858), which is recruiting participants at the moment.

BTK inhibition might provide therapeutic benefit in amyotrophic lateral sclerosis (ALS), a fatal neurodegenerative disease, whose pathophysiology is thought to be multifactorial, involving neuroinflammation, oxidative stress, disrupted protein homeostasis, mitochondrial dysfunction, and glutamate excitotoxicity. In SOD1 ^G93A^ mice, ibrutinib, administered orally either prophylactically or therapeutically, significantly delayed symptom onset, extended survival time, and improved motor function[91]. It also reduced muscular atrophy, necrosis, and pro-inflammatory cytokine levels while decreasing ionized calcium-binding adapter molecule 1 (IBA-1) and glial fibrillary acidic protein (GFAP) expression. These effects were associated with modulation of the mTOR/Akt/PI3K signaling pathway in the medulla, motor cortex, and spinal cord. These findings should be scrutinized in the clinical trials.

In models of stress, BTK has been shown to be upregulated in brain regions associated with stress and anxiety. Interestingly, BTK phosphorylation (pBTK) in response to stress is induced more in female hippocampi and amygdala compared to male animals, although the baseline levels of pBTK before stress inducement are the same in both sexes in both brain regions, suggesting a sex-specific role of BTK in mediating adverse responses following adverse stimuli [59,92].

Findings on BTKi in other diseases are summarized in Table 2.

## Outlook and conclusive remarks

Therapeutic targeting of BTK by its inhibitors in inflammatory CNS disorders or treatment of autoimmune diseases is an emerging strategy that holds huge potential and can support current treatments of several neurological disorders and bring new paradigms toward brain disease. However, selectivity is the major concern for BTK inhibitors; the second generation of BTKi may cause much fewer adverse effects than the first generation, but there is still much work to be done on safety issues. One of the most often reported adverse effects of ibrutinib and typically one of the top three reasons for calling for better selectivity in BTKi is atrial fibrillation, which is also another reason for discontinuation of ibrutinib. The development of irreversible BTKi has advanced more quickly than that of reversible inhibitors. As had been shown in the treatment with ibrutinib, covalent, irreversible inhibition does in fact raise the likelihood of off-target reactivity to biomolecules, which may result in immunotoxicity, mutagenicity, and hepatotoxicity. Nevertheless, irreversible inhibitors demonstrate greater efficacy and resilience against pharmacokinetic issues than reversible inhibitors due to the establishment of a permanent covalent bond. Nevertheless, the recent findings on BTK and the efficacy of BTKi in neurological diseases, especially in MS and AD, may be a promising start for these molecules to become potential candidates in neurological disease targeting.

## Figures and Tables

**Table 1:** BTK inhibitors (BTKi) approved or currently being investigated in clinical trials.

Compound	No of Clinical Trials	Target Disease	Mode of inhibition
**Irreversible**			

*ibrutinib*	404 (83 recruiting, 116 completed, 33 terminated, 18 withdrawn, 13 not yet recruiting	CLL, GvHD, MCL, MZL, WM	Covalent (Cys-481)
*acalabrutinib*	149 (65 recruiting, 26 completed, 5 terminated, 5 withdrawn)	CLL, MCL	Covalent (Cys-481)
*zanubrutinib*	95 (49 recruiting, 21 completed, 14 not yet recruiting)	MCL	Covalent (Cys-481)
*tirabrutinib*	11 (2 recruiting, 6 completed, 1 withdrawn)	LPL, PCNSL, WM	Covalent (Cys-481)
*branebrutinib*	8 (7 completed)	SS, SLE	Covalent (Cys-481)
*remIbrutinib*	10 (3 recruiting, 1 terminated)	CSU, SS	Covalent (Cys-481)
*evobrutinib*	18 (11 completed, 1 recruiting, 3 terminated)	MS	Covalent (Cys-481)
*tolebrutinib*	9 (2 completed, 1 recruiting)	MS	Covalent (Cys-481)

**Reversible**			

*BMS-986142*	7 (6 completed, 1 terminated)	RA	Reversible SH3 domain
*rilzabrutinib*	9 (2 completed, 6 recruiting, 1 terminated)	ITP, Pemphigus	Reversible SH3 domain, transient covalent (Cys-481)
*BIIB068*	1 (completed)	SLE	Reversible SH3 domain
*SHR1459*	11 (2 recruiting, 3 completed, 1 withdrawn, 4 not recruiting)	Different autoimmune diseases, Lymphoma	Reversible SH3 domain
*fenebrutinib/GDC0853*	9 (4 completed, 1 terminated, 4 recruiting)	LL, Lymphoma, Progressive MS, RMS, SLE, Urticaria	Reversible SH3 domain

CLL: Chronic lymphocytic leukemia; CSU: Chronic spontaneous urticaria; GvHD: Graft-versus-host disease; ITP: Immune thrombocytopenia; LL: Lymphocytic Leukemia; LPL: Lymphoplasmacytic lymphoma; MCL: Mantle cell lymphoma; MS: Multiple sclerosis; MZL: Marginal zone lymphoma; PCNSL: Primary central nervous system lymphoma; RA: Rheumatoid arthritis; RMS: Rhabdomyosarcoma; SLE: Systemic lupus erythematosus; SS: Sjögren syndrome; WM: Waldenström macroglobulinemia

**Table 2:** Summary of findings on clinical and preclinical efficacy of BTK inhibitors in neurological diseases.

Clinical findings			

Disease	Disease type	Clinical finding(s)	Ref(s)
*Multiple sclerosis*	RRMS	Tolebrutinib and evobrutinib reduced acute inflammation lesions. 66 % of patients experienced adverse effects after being treated with 75 mg/day evobrutinib.	[5,95]
	SPMS	Tolebrutinib reduced the gandolinium enhancing lesions. 54 % patients experienced adverse effects, but these were not dose dependent.	[95]
	PPMS	Fenebrutinib trial ongoing	NCT04544449 (clinicaltrials.gov)

**Preclinical findings**			

**Disease**	**Preclinical finding(s)**	**Associated mechanism(s)**	**Ref(s)**

*Aging*	BTK was overexpressed in Zmpste24−/− mice (model of aging mice). Ibrutinib reduced anxiety-like behavior and memory loss in mice.	Ibrutinib decreased the levels of p53, p21, and p16 (senescence markers).	[73]
*Alzheimer’s disease*	BTK transcript and protein expressions were increased in AD brains and were associated with synaptic loss. Ibrutinib improved long-term memory in 5xFAD model of AD.	Ibrutinib decreased tau phosphorylation and regulated microglial phagocytosis.	[50,51,96]
*Brain injury*	Ibrutinib reduced the size of cerebral infarcts, improved neurological deficits, and mitigated pathological alterations.	Ibrutinib increased significantly autophagy by regulating the PI3K/AKT/mTOR pathway.	[56,57]
*PCNSL*	The exceptional effectiveness of ibrutinib as a standalone treatment in patients with PCNSL that has recurred or does not respond to other treatments.	Blocking of BTK and PI3K/mTOR pathways enhanced the effectiveness of ibrutinib treatment in cases of human PCNSLs with CD79B mutations.	[82]
*Stress*	Inhibition of BTK effectively decreased the anxious behavior observed in mice subjected to stress.	BTKi attenuated the induction of NLRP3 inflammasome, Caspase 1 and IL1β.	[59]
*Multiple sclerosis*	BTKi showed positive effects in remyelination both in vivo and ex vivo models.	BTKi inhibited B-cell function and promoted anti-inflammatory macrophage differentiation.	[97]
*ALS*	In SOD1 G93A mice, BTKi significantly delayed symptom onset, extended survival time, and improved motor function.	BTKi reduced muscular atrophy, necrosis, and pro-inflammatory cytokine levels and decreased IBA-1 and GFAP expression. And modulation of mTOR/Akt/PI3K signaling pathway.	[91]
*NMOSD*	BTKi mitigated demyelination, edema, axonal injury, and astrocyte–microglia interaction.	Reduced B-cell activation, maturation, and aquaporin-4 autoantibody production were observed. Transcriptome analysis showed BTKi downregulated chemokine-related genes and genes associated with cell adhesion and migration in microglia.	[90]

AD = Alzheimer’s disease; ALS = BTKi = inhibitors of Bruton’s tyrosine kinase; GFAP = glial fibrillary acidic protein; IBA-1 = ionized calcium-binding adapter molecule 1; NLRP3 = Nucleotide-binding oligomerization domain 3; NMOSD = neuromyelitis optica spectrum disorder; PCNSL = primary central nervous system lymphoma; RRMS = relapsing-remitting multiple sclerosis; SPMS = secondary progressive multiple sclerosis

## References

[1] Wu J, Liu C, Tsui ST (2016). Second-generation inhibitors of Bruton tyrosine kinase. J Hematol Oncol.

[2] (2021). Estupiñán HY, Berglöf A, Zain R, et al. Front Cell Dev Biol.

[3] Jongstra-Bilen J, Puig Cano A, Hasija M (2008). Dual Functions of Bruton’s Tyrosine kinase and tec kinase during fcγ receptor-induced signaling and phagocytosis. J Immunol.

[4] Ringheim GE, Wampole M, Oberoi K (2021). Bruton’s Tyrosine Kinase (BTK) Inhibitors and Autoimmune Diseases: Making Sense of BTK Inhibitor Specificity Profiles and Recent Clinical Trial Successes and Failures. Front Immunol.

[5] Montalban X, Arnold DL, Weber MS (2019). Placebo-Controlled Trial of an Oral BTK Inhibitor in Multiple Sclerosis. N Engl J Med.

[6] Smith CIE, Burger JA (2021). Resistance Mutations to BTK Inhibitors Originate From the NF-κB but Not From the PI3K-RAS-MAPK Arm of the B Cell Receptor Signaling Pathway. Front Immunol.

[7] Wen T, Wang J, Shi Y (2021). Inhibitors targeting Bruton’s tyrosine kinase in cancers: drug development advances. Leukemia.

[8] Tasso B, Spallarossa A, Russo E (2021). The Development of BTK Inhibitors: A Five-Year Update. Molecules.

[9] Dolgin E (2021). BTK blockers make headway in multiple sclerosis. Nat Biotechnol.

[10] Zhang D, Gong H, Meng F (2021). Recent Advances in BTK Inhibitors for the Treatment of Inflammatory and Autoimmune Diseases. Molecules.

[11] Woyach JA, Furman RR, Liu T-M (2014). Resistance Mechanisms for the Bruton’s Tyrosine Kinase Inhibitor Ibrutinib. N Engl J Med.

[12] Young WB, Barbosa J, Blomgren P (2016). Discovery of highly potent and selective Bruton’s tyrosine kinase inhibitors: Pyridazinone analogs with improved metabolic stability. Bioorg Med Chem Lett.

[13] Noy A, de Vos S, Thieblemont C (2017). Targeting Bruton tyrosine kinase with ibrutinib in relapsed/refractory marginal zone lymphoma. Blood.

[14] Honigberg LA, Smith AM, Sirisawad M (2010). The Bruton tyrosine kinase inhibitor PCI-32765 blocks B-cell activation and is efficacious in models of autoimmune disease and B-cell malignancy. Proc Natl Acad Sci U S A.

[15] Wang ML, Rule S, Martin P (2013). Targeting BTK with ibrutinib in relapsed or refractory mantle-cell lymphoma. N Engl J Med.

[16] Byrd JC, Furman RR, Coutre SE (2013). Targeting BTK with ibrutinib in relapsed chronic lymphocytic leukemia. N Engl J Med.

[17] Treon SP, Tripsas CK, Meid K (2015). Ibrutinib in previously treated Waldenström’s macroglobulinemia. N Engl J Med.

[18] Miklos D, Cutler CS, Arora M (2017). Ibrutinib for chronic graft-versus-host disease after failure of prior therapy. Blood.

[19] Levade M, David E, Garcia C (2014). Ibrutinib treatment affects collagen and von Willebrand factor-dependent platelet functions. Blood.

[20] Shanafelt TD, Wang XV, Kay NE (2019). Ibrutinib-Rituximab or Chemoimmunotherapy for Chronic Lymphocytic Leukemia. N Engl J Med.

[21] Munir T, Brown JR, O’Brien S (2019). Final analysis from RESONATE: Up to six years of follow-up on ibrutinib in patients with previously treated chronic lymphocytic leukemia or small lymphocytic lymphoma. Am J Hematol.

[22] Nicolson PLR, Hughes CE, Watson S (2018). Inhibition of Btk by Btk-specific concentrations of ibrutinib and acalabrutinib delays but does not block platelet aggregation mediated by glycoprotein VI. Haematologica.

[23] Tam CS, Opat S, D’Sa S (2020). A randomized phase 3 trial of zanubrutinib vs ibrutinib in symptomatic Waldenström macroglobulinemia: the ASPEN study. Blood.

[24] Sharman JP, Egyed M, Jurczak W (2020). Acalabrutinib with or without obinutuzumab versus chlorambucil and obinutuzmab for treatment-naive chronic lymphocytic leukaemia (ELEVATE TN): a randomised, controlled, phase 3 trial. Lancet.

[25] Ghia P, Pluta A, Wach M (2020). ASCEND: Phase III, Randomized Trial of Acalabrutinib Versus Idelalisib Plus Rituximab or Bendamustine Plus Rituximab in Relapsed or Refractory Chronic Lymphocytic Leukemia. J Clin Oncol.

[26] Hillmen P, Eichhorst B, Brown JR (2023). Zanubrutinib Versus Ibrutinib in Relapsed/Refractory Chronic Lymphocytic Leukemia and Small Lymphocytic Lymphoma: Interim Analysis of a Randomized Phase III Trial. JCO.

[27] Bye AP, Unsworth AJ, Desborough MJ (2017). Severe platelet dysfunction in NHL patients receiving ibrutinib is absent in patients receiving acalabrutinib. Blood Adv.

[28] Ma B, Metrick CM, Gu C (2022). Optimization of a novel piperazinone series as potent selective peripheral covalent BTK inhibitors. Bioorganic & Medicinal Chemistry Letters.

[29] Elamin G, Aljoundi A, Alahmdi MI (2022). Battling BTK mutants with noncovalent inhibitors that overcome Cys481 and Thr474 mutations in Waldenström macroglobulinemia therapy: structural mechanistic insights on the role of fenebrutinib. J Mol Model.

[30] Brullo C, Villa C, Tasso B (2021). Btk Inhibitors: A Medicinal Chemistry and Drug Delivery Perspective. Int J Mol Sci.

[31] Dickson EJ, Hille B (2019). Understanding phosphoinositides: rare, dynamic, and essential membrane phospholipids. Biochemical Journal.

[32] Solvason N, Wu WW, Kabra N (1998). Transgene Expression of bcl-xL Permits Anti-immunoglobulin (Ig)-induced Proliferation in xid B Cells. Journal of Experimental Medicine.

[33] Schaeffer EM, Debnath J, Yap G (1999). Requirement for Tec Kinases Rlk and Itk in T Cell Receptor Signaling and Immunity. Science.

[34] Smith CIE, Islam TC, Mattsson PT (2001). The Tec family of cytoplasmic tyrosine kinases: mammalian Btk, Bmx, Itk, Tec, Txk and homologs in other species. Bioessays.

[35] Basile N, Danielian S, Oleastro M (2009). Clinical and Molecular Analysis of 49 Patients With X-linked Agammaglobulinemia From A Single Center in Argentina. J Clin Immunol.

[36] Väliaho J, Smith CIE, Vihinen M (2006). BTKbase: the mutation database for X-linked agammaglobulinemia. Hum Mutat.

[37] Rawlings DJ, Saffran DC, Tsukada S (1993). Mutation of Unique Region of Bruton’s Tyrosine Kinase in Immunodeficient XID Mice. Science.

[38] Cancro MP, Sah AP, Levy SL (2001). xid mice reveal the interplay of homeostasis and Bruton’s tyrosine kinase-mediated selection at multiple stages of B cell development. International Immunology.

[39] Torke S, Pretzsch R, Häusler D (2020). Inhibition of Bruton’s tyrosine kinase interferes with pathogenic B-cell development in inflammatory CNS demyelinating disease. Acta Neuropathol.

[40] Middendorp S, Dingjan GM, Hendriks RW (2002). Impaired Precursor B Cell Differentiation in Bruton’s Tyrosine Kinase-Deficient Mice. The Journal of Immunology.

[41] Cariappa A, Tang M, Parng C (2001). The Follicular versus Marginal Zone B Lymphocyte Cell Fate Decision Is Regulated by Aiolos, Btk, and CD21. Immunity.

[42] Lünemann JD, Malhotra S, Shinohara ML (2021). Targeting Inflammasomes to Treat Neurological Diseases. Ann Neurol.

[43] Rip J, Van Der Ploeg EK, Hendriks RW (2018). The Role of Bruton’s Tyrosine Kinase in Immune Cell Signaling and Systemic Autoimmunity. Crit Rev Immunol.

[44] DiSabato D, Quan N, Godbout JP (2016). Neuroinflammation: The Devil is in the Details. J Neurochem.

[45] Chen F, Ghosh A, Lin J (2020). 5-lipoxygenase pathway and its downstream cysteinyl leukotrienes as potential therapeutic targets for Alzheimer’s disease. Brain Behav Immun.

[46] Zheng Z-H, Tu J-L, Li X-H (2021). Neuroinflammation induces anxiety- and depressive-like behavior by modulating neuronal plasticity in the basolateral amygdala. Brain Behav Immun.

[47] Subramanian J, Savage JC, Tremblay M-È (2020). Synaptic Loss in Alzheimer’s disease: mechanistic insights provided by two-photon in vivo imaging of transgenic mouse models. Frontiers in Cellular Neuroscience [Internet].

[48] Zhang Y, Chen K, Sloan SA (2014). An RNA-Sequencing transcriptome and splicing database of glia, neurons, and vascular cells of the cerebral cortex. J Neurosci.

[49] Zhang Y, Sloan SA, Clarke LE (2016). Purification and characterization of progenitor and mature human astrocytes reveals transcriptional and functional differences with mouse. Neuron.

[50] Keaney J, Gasser J, Gillet G (2019). Inhibition of Bruton’s tyrosine kinase modulates microglial phagocytosis: therapeutic implications for Alzheimer’s disease. J Neuroimmune Pharmacol.

[51] Lee H, Jeon SG, Kim J (2021). Ibrutinib modulates Aβ/tau pathology, neuroinflammation, and cognitive function in mouse models of Alzheimer’s disease. Aging Cell [Internet].

[52] Dybowski S, Torke S, Weber MS (2023). Targeting B cells and microglia in multiple sclerosis with bruton tyrosine kinase inhibitors: a review. JAMA Neurol.

[53] Fusco R, Siracusa R, Genovese T (2020). Focus on the role of NLRP3 inflammasome in diseases. Int J Mol Sci.

[54] Guo H, Callaway JB, Ting JP-Y (2015). Inflammasomes: mechanism of action, role in disease, and therapeutics. Nat Med.

[55] Youm Y-H, Grant RW, McCabe LR (2013). Canonical Nlrp3 inflammasome links systemic low grade inflammation to functional decline in aging. Cell Metab.

[56] Jin L, Mo Y, Yue E-L (2021). Ibrutinib ameliorates cerebral ischemia/reperfusion injury through autophagy activation and PI3K/Akt/mTOR signaling pathway in diabetic mice. Bioengineered.

[57] Ito M, Shichita T, Okada M (2015). Bruton’s tyrosine kinase is essential for NLRP3 inflammasome activation and contributes to ischaemic brain injury. Nat Commun.

[58] Bittner ZA, Liu X, Mateo Tortola M (2021). BTK operates a phospho-tyrosine switch to regulate NLRP3 inflammasome activity. J Exp Med.

[59] Ghosh S, Mohammed Z, Singh I (2021). Pharmacological Inhibition of BTK reduces neuroinflammation and stress induced anxiety in vivo [Internet]. bioRxiv.

[60] Franke M, Bieber M, Kraft P (2021). The NLRP3 inflammasome drives inflammation in ischemia/reperfusion injury after transient middle cerebral artery occlusion in mice. Brain Behav Immun.

[61] Ghosh S, Mohammed Z, Singh I (2021). Bruton’s tyrosine kinase drives neuroinflammation and anxiogenic behavior in mouse models of stress. J Neuroinflammation.

[62] Cui Y, Yu H, Bu Z (2022). Focus on the Role of the NLRP3 Inflammasome in Multiple Sclerosis: Pathogenesis, Diagnosis, and Therapeutics. Front Mol Neurosci.

[63] Hanslik KL, Ulland TK (2020). The Role of Microglia and the Nlrp3 Inflammasome in Alzheimer’s Disease. Front Neurol.

[64] Lassmann H (2018). Pathogenic mechanisms associated with different clinical courses of multiple sclerosis. Front Immunol.

[65] Milo R (2019). Therapies for multiple sclerosis targeting B cells. Croat Med J.

[66] Touil H, Li R, Zuroff L (2023). Cross-talk between B cells, microglia and macrophages, and implications to central nervous system compartmentalized inflammation and progressive multiple sclerosis. eBioMedicine.

[67] Comi G, Bar-Or A, Lassmann H (2021). Role of B cells in multiple sclerosis and related disorders. Ann Neurol.

[68] Li R, Tang H, Burns JC (2022). BTK inhibition limits B-cell-T-cell interaction through modulation of B-cell metabolism: implications for multiple sclerosis therapy. Acta Neuropathol.

[69] Pellerin K, Rubino SJ, Burns JC (2021). MOG autoantibodies trigger a tightly-controlled FcR and BTK-driven microglia proliferative response. Brain.

[70] Hopkins BT, Bame E, Bajrami B (2022). Discovery and preclinical characterization of BIIB091, a reversible, selective BTK inhibitor for the treatment of multiple sclerosis. J Med Chem.

[71] Saez-Atienzar S, Masliah E (2020). Cellular senescence and Alzheimer disease: the egg and the chicken scenario. Nat Rev Neurosci.

[72] Peters R (2006). Ageing and the brain. Postgrad Med J.

[73] Ekpenyong-Akiba AE, Poblocka M, Althubiti M (2020). Amelioration of age-related brain function decline by Bruton’s tyrosine kinase inhibition. Aging Cell.

[74] Mendez MF (2021). The Relationship Between Anxiety and Alzheimer’s Disease. J Alzheimer’s Dis Rep.

[75] Limorenko G, Lashuel HA (2022). Revisiting the grammar of Tau aggregation and pathology formation: how new insights from brain pathology are shaping how we study and target Tauopathies. Chem Soc Rev.

[76] Schaff LR, Grommes C (2022). Primary central nervous system lymphoma. Blood.

[77] Ngo VN, Young RM, Schmitz R (2011). Oncogenically active MYD88 mutations in human lymphoma. Nature.

[78] Schaff LR, Grommes C (2021). Update on novel therapeutics for primary CNS lymphoma. Cancers.

[79] Goldwirt L, Beccaria K, Ple A (2018). Ibrutinib brain distribution: a preclinical study. Cancer Chemother Pharmacol.

[80] Lionakis MS, Dunleavy K, Roschewski M (2017). Inhibition of B cell receptor signaling by Ibrutinib in primary CNS lymphoma. Cancer Cell.

[81] Zhai Y, Zhou X, Wang X (2022). Novel insights into the biomarkers and therapies for primary central nervous system lymphoma. Ther Adv Med Oncol.

[82] Grommes C, Pastore A, Palaskas N (2017). Ibrutinib unmasks critical role of bruton tyrosine kinase in primary CNS lymphoma. Cancer Discovery.

[83] Soussain C, Choquet S, Blonski M (2019). Ibrutinib monotherapy for relapse or refractory primary CNS lymphoma and primary vitreoretinal lymphoma: Final analysis of the phase II ‘proof-of-concept’ iLOC study by the Lymphoma study association (LYSA) and the French oculo-cerebral lymphoma (LOC) network. European Journal of Cancer.

[84] Grommes C, Tang SS, Wolfe J (2019). Phase 1b trial of an ibrutinib-based combination therapy in recurrent/refractory CNS lymphoma. Blood.

[85] Houillier C, Chabrot CM, Moles-Moreau M-P (2021). Rituximab-Lenalidomide-Ibrutinib combination for relapsed/refractory primary CNS lymphoma: A Case Series of the LOC Network. Neurology.

[86] Munakata W, Tobinai K (2021). Tirabrutinib hydrochloride for B-cell lymphomas. Drugs Today.

[87] Okita Y, Kano-Fujiwara R, Nakatsuka S-I (2021). Histological verification of the treatment effect of tirabrutinib for relapsed/refractory primary central nervous system lymphoma. Exp Hematol Oncol.

[88] Yang C, Cui Y, Ren X (2022). Orelabrutinib combined with Lenalidomide and immunochemotherapy for relapsed/refractory primary central nervous system lymphoma: a retrospective analysis of case series. Front Oncol.

[89] Yu CG, Bondada V, Iqbal H (2021). Inhibition of Bruton Tyrosine Kinase Reduces Neuroimmune Cascade and Promotes Recovery after Spinal Cord Injury. Int J Mol Sci.

[90] Liu Y, Huang Z, Zhang T-X (2023). Bruton’s tyrosine kinase-bearing B cells and microglia in neuromyelitis optica spectrum disorder. J Neuroinflammation.

[91] Zheng C, Li W, Ali T (2023). Ibrutinib Delays ALS Installation and Increases Survival of SOD1G93A Mice by Modulating PI3K/mTOR/Akt Signaling. J Neuroimmune Pharmacol.

[92] Li K, Ran B, Wang Y (2022). PLCγ2 impacts microglia-related effectors revealing variants and pathways important in Alzheimer’s disease. Front Cell Dev Biol.

[93] Vastrad B, Vastrad C (2023). Screening of the key genes and signaling pathways for schizophrenia using bioinformatics and next generation sequencing data analysis [Internet].

[94] Roussou IG, Papanikolopoulou K, Savakis C (2019). Drosophila Bruton’s tyrosine kinase regulates habituation latency and facilitation in distinct mushroom body neurons. J Neurosci.

[95] Reich DS, Arnold DL, Vermersch P (2021). Safety and efficacy of tolebrutinib, an oral brain-penetrant BTK inhibitor, in relapsing multiple sclerosis: a phase 2b, randomised, double-blind, placebo-controlled trial. Lancet Neurol.

[96] Mali AS, Novotny J (2022). Opioid receptor activation suppresses the neuroinflammatory response by promoting microglial M2 polarization. Mol Cell Neurosci.

[97] Martin E, Aigrot M-S, Grenningloh R (2020). Bruton’s Tyrosine Kinase Inhibition Promotes Myelin Repair. Brain Plast.

